# University of the State of New York

**Published:** 1911-07

**Authors:** 


### University of the State of New York

### Changes in the State Board of Medical Examiners

At a meeting of the Regents held at Albany, April 19, 1911, the following appointments were recommended to fill vacancies in the board:

For examiner in surgery, Arthur W. Booth, of Elmira, term to expire July 31, 1912.

For examiner in obstetrics and gynecology, Aaron B. Miller of Syracuse, term to expire July 31, 1912.

For examiner in bacteriology, Thomas B. Carpenter, of Buffalo, term to expire July 31, 1913.

For examiner in anatomy, Henry B. Minton, of Brooklyn, for a term of three years.

For examiner in chemistry, for a term of three years, Floyd S. Farnsworth, of Plattsburg, to succeed himself.

For examiner in physiology, for a term of three years, Ralph H. Williams, of Rochester, to succeed himself.

The other members of the board serving unexpired terms are: Lee H. Smith, of Buffalo, examiner in pathology; William H. Park, of New York, examiner in hygiene and sanitation; Glentworth R. Butler, of Brooklyn, examiner in diagnosis.

Dr. Maurice J. Lewi, of New York, is the secretary of the board.

